# Association between the Frequency of Protein-Rich Food Intakes and Kihon-Checklist Frailty Indices in Older Japanese Adults: The Kyoto-Kameoka Study

**DOI:** 10.3390/nu10010084

**Published:** 2018-01-13

**Authors:** Miwa Yamaguchi, Yosuke Yamada, Hinako Nanri, Yoshizu Nozawa, Aya Itoi, Eiichi Yoshimura, Yuya Watanabe, Tsukasa Yoshida, Keiichi Yokoyama, Chiho Goto, Kazuko Ishikawa-Takata, Hisamine Kobayashi, Misaka Kimura, Kyoto-Kameoka Study Group

**Affiliations:** 1 Department of Nutrition and Metabolism, National Institutes of Biomedical Innovation, Health and Nutrition, Tokyo 162-8636, Japan; yamaday@nibiohn.go.jp (Y.Y.); kimurahinako0319@gmail.com (H.N.); t-yoshida@nibiohn.go.jp (T.Y.); 2 Laboratory of Applied Health Sciences, Kyoto Prefectural University of Medicine, Kyoto 602-8566, Japan; yuwatana@mail.doshisha.ac.jp (Y.W.); misaka@kyotogakuen.ac.jp (M.K.); 3 Department of Health and Sports Sciences, Kyoto Gakuen University, Kyoto 621-8555, Japan; 4 Ajinomoto Co., Inc., Tokyo 210-8681, Japan; yoshizu_nozawa@ajinomoto.com (Y.N.); hisamine_kobayashi@ajinomoto.com (H.K.); 5 Department of Health, Sports and Nutrition, Faculty of Health and Welfare, Kobe Women’s University, Hyogo 650-0046, Japan; aitoi@kwjc.kobe-wu.ac.jp; 6 Department of Food and Health Sciences, Prefectural University of Kumamoto, Kumamoto 862-8502, Japan; eyoshi@pu-kumamoto.ac.jp; 7 Faculty of Health and Sports Science, Doshisha Unviersity, Kyoto 610-0394, Japan; 8 Senior Citizen’s Welfare Section, Kameoka City Government, Kyoto 621-8501, Japan; 9 Department of Business Administration, Kyoto Gakuen University, Kyoto 621-8555, Japan; physiol.yokoyama@gmail.com; 10 Department of Health and Nutrition, Faculty of Health and Human Life, Nagoya Bunri University, Aichi 492-8520, Japan; goto.chiho@nagoya-bunri.ac.jp; 11 Department of Nutritional Epidemiology and Shokuiku, National Institutes of Biomedical Innovation, Health and Nutrition, Tokyo 162-8636, Japan; kazu@nibiohn.go.jp

**Keywords:** frailty, Kihon-Checklist, frequency of protein-rich food, seafood, dairy products

## Abstract

We aimed to investigate whether frequencies of protein-rich food intake were associated with frailty among older Japanese adults. A cross-sectional study was conducted in 2011 among 3843 men and 4331 women in a population-based cohort of Kameoka city, Kyoto Prefecture, Japan. Frailty was assessed by the weighted score based on the 25-item Kihon-Checklist. The frequency of protein-rich food intake was examined as “seafood”, “meat”, “dairy products”, “eggs”, and “soy products”. The outcome of frailty was analyzed with a multiple logistic regression model using the frequency of protein-rich food intake. When compared to the first quartile, it was observed that there was a significant association between the lower adjusted prevalence ratio (PR) for frailty and the frequency of seafood intake in the fourth quartile among men (PR 0.64, 95% confidence interval (CI), 0.42, 0.99) and from the second quartile to the third quartile among women (PR 0.61, 95% CI, 0.43, 0.85; PR 0.64, 95% CI, 0.46, 0.91). The frequency of dairy products intake in the third quartile among women was significantly associated with a lower PR for frailty (*p*-value = 0.013). Our findings suggest that the consumption of seafood and dairy products may help older adults in maintaining their independence.

## 1. Introduction

It is estimated that Japan has the highest proportion of elderly citizens globally. The Japanese Census Bureau approximates that, as of 2015, 27% of the Japanese population is aged 65 years and above [1]. The number of monthly users of long-term care (LTC) insurance services has also dramatically increased over the past few decades in Japan [1]. In light of this, an optimal dietary protein intake has been proposed to help older adults maintain a higher level of physical and cognitive function, which consequently affects the ability to maintain independence and have a healthy lifespan, as well as lower the rate of falls and mortality [2,3,4]. In a recent review article, three studies that included older Japanese populations reported on the significant association between higher protein intake and a lower prevalence of frailty; however, there was a null association in two other studies [5]. Therefore, it is still controversial whether there is a significant association between protein-rich food intake (e.g., seafood, meat, dairy products, egg, and soy products) and frailty status in older adults. In addition, previous studies have mostly reported on the association between protein-rich food intake and either physical or psychological frailty [6,7,8,9,10,11].

A holistic assessment of frailty is required to investigate independent living and the link to protein-rich food intake among older adults. Consequently, we designed the present research as described below. First, the present study measured the frequency of consumption of five types of protein-rich foods (i.e., seafood, meat, dairy products, egg, and soy products) that we collected and were referenced by the Japanese food guide [12]. Second, we employed a well-assessed indicator of frailty. Frailty is defined by the phenotype of increased vulnerability due to aging and disabilities of physiologic systems [13]. The scale for a physically frail phenotype based on Fried’s definition is used broadly [14,15]. However, the current Japanese LTC insurance system uses the 25-item Kihon-Checklist (KCL) as a self-reported questionnaire to screen for frailty among community-dwelling older residents [16,17]. The KCL has been recently validated in various studies to screen for future risks of LTC certification, and has been translated to other languages for use in other countries [18,19]. The strength of the KCL lies in the fact that apart from calculating the total frailty score, it can also assess the seven subdomains, including physical and psychological assessments, related to frailty and the risks of future care needs.

Thus, the aim of this study was to clarify the association between the frequency of consumption of five types of protein-rich foods and frailty assessed by the KCL for independent living among older Japanese adults.

## 2. Materials and Methods 

### 2.1. Study Participants

The design of the current survey has been described previously [20]. We recruited 16,474 adults aged 65 years and above who resided in Kameoka city in July 2011 and who did not receive LTC or LTC insurance. Among them, 12,054 responded to a postal survey (response rate: 73.2%). In February 2012, an additional survey, which included the dietary questionnaire, was conducted for 11,985 of the 12,054 residents, after eliminating 69 people who died; the number of respondents was 8370 (response rate: 69.8%). We excluded 51 cases for which we were not able to accurately match the individuals or for which there was an inconsistency of sex between the baseline and the additional survey due to an error in reassignments (e.g., a mistake of distribution or type error). An additional 123 cases were missing all of the food frequencies and were excluded from this study. From the remaining 8196 residents, we further excluded the data of 22 people who exceeded ±4 standard deviations (SD) of the average of total energy intake among this population (+4 SD: 4020 kcal/day in men; 2975 kcal/day in women) as implausible data of food frequency; no participants exceeded −4 SD of the average total energy intake. The final number of eligible participants in this study was 8174 (3843 men and 4331 women). All participants gave informed consent prior to participation in this study with a response to the questionnaire. A detailed version of the ethical issues was described in our previous study [20], and the entire study protocol was reviewed and approved by the Ethics Committee of the Kyoto Prefectural University of Medicine (RBMR-E-363) and the National Institute of Health and Nutrition (NIHN187-3).

### 2.2. Frequency of Protein-Rich Food Intake

The participants responded to the frequency of protein-rich food consumption using the commonly used Japanese food frequency questionnaire (FFQ) and the additional survey to collect dietary habits and more information, such as demographics and lifestyle profiles [21,22,23,24]. The participants responded to questions about their average intake frequencies of 46 food items and beverages over the past year. We classified protein-rich foods into five groups according to the Japanese general diet [12]: (1) seafood: fish, bone-edible small fish, canned tuna, cuttlefish, squid, octopus, shrimp, crab, shellfish, and fish paste products; (2) meat: chicken, beef, pork, liver, ham, sausage, bacon, and salami-sausage; (3) dairy products: milk and yogurt; (4) egg: principally poultry egg; and (5) soy products: miso soup, tofu (soybean curd), fried tofu paste, and fried tofu. The frequency of food intake was categorized into the following eight categories: (1) almost none, (2) 1–3 times/month, (3) 1–2 times/week, (4) 3–4 times/week, (5) 5–6 times/week, (6) once a day, (7) twice a day, and (8) ≥3 times/day. In this analysis, we divided these categories into 0, 0.1, 0.2, 0.5, 0.8, 1.0, 2.0, and 3.0 times/day, respectively. These frequencies were then summed up separately by the five types of protein-rich foods (Table 1). We tested the reproducibility of the frequency of protein-rich food intake, between February and September 2012, for the correlation among 227 men and 221 women (Table 1).

### 2.3. Definition of Frailty

The KCL is a unique screening test to assess not only an overall frail score but also subdomain frail components of physiological (Sections 1–4), psychological (Sections 5–7), and social (Sections 1 and 5) aspects. The KCL consists of 25 items and has seven sections: (1) instrumental activities of daily living (IADL), (2) physical function/strength, (3) malnutrition, (4) oral function, (5) socialization/housebound, (6) memory, and (7) mood [18]. When the response (Yes or No) indicates a risk of frailty, a +1 score is given. For example, a “no” response to the question “Do you go out by bus or train by yourself?” would increase the score by one point. Higher KCL scores (on a scale of 0–25) indicate severely impaired functioning and severe frailty. A total KCL score of ≥7 points suggests frailty with a higher risk of requiring long-term care [19,25,26].

We assessed the missing data of the KCL score that was “missing not at random”. Since there was a possibility that the missing data of the KCL score was related to the risk of frailty, we decided to predict the missing score from a regression model as a conditional mean imputation [27,28]. The total weighted score of the KCL (total β score) ranged from 0 to 10.69 with 23 items due the lack of scores for two specific items in the sections ‘oral function’ and ‘mood’. After conducting a receiver operating characteristic (ROC) curve analysis, we decided on the 75th percentile in the total β score as the cutoff point for defining frailty in this study, which was 3.80. The details for developing the model are described in the Appendix A and Table A1.

### 2.4. Covariates

Because frailty results from aging-associated decline in reserve and function [13], age (years) is a primary covariate to consider. The present age was calculated from the date of birth on the resident register in the city office. Other possible variables related to both food intake frequency and frailty were as follows: family structure (living alone or living with someone/others), educational attainment (≤9, 10–12, and ≥13 years), self-rated economic conditions (good or poor), diet supplement use (yes or no), diet treatment (yes or no), current smoking (yes, past, or never), body mass index (BMI) (<18.5, 18.5–24.9, and ≥25 kg/m^2^), total energy intake (kcal/day) calculated with the food frequency questionnaire, and population density (province <1000 or city ≥1000 people/km^2^). The population density was employed for adjusting the different seven areas in Kameoka city based on the geographical, socioeconomic, or other diet-related difference between urban and rural areas [29]. The BMI (kg/m^2^) was calculated by dividing the weight (kg) by the square of the height (m^2^). The total energy intake (kcal/day) was ascertained using the FFQ. We did not use the physical activity variable as a covariate, since the KCL included physical activity status in the IADL, physical function/strength, and socialization/housebound components.

### 2.5. Statistical Analysis

The difference between non-frailty and frailty in each covariate was evaluated using the chi-square test for categorical variables or the unpaired *t*-test for continuous variables among each sex. To examine the associations of the intake frequencies of the aforementioned five types of protein-rich foods with the prevalence of frailty (total β score: ≥3.80 or <3.80), multiple logistic regression models were fitted by adjusting for all the possible covariates in Model 1. Model 2 consisted of Model 1 with the addition of all the types of food intake frequencies. The number of samples in Model 1 (3843 men and 4331 women) was different from that in Model 2 (2807 men and 2978 women) because we recruited participants who had responded to at least one of the five types of protein-rich foods. To compare results between Model 1 and Model 2 with the same number of participants, we analyzed data from the participants who had responded to all the food type frequencies.

A multiple regression model was performed to estimate the association of the β score of each of the six sections of the KCL (i.e., IADL, physical function, oral function, socialization, memory, and mood) with the frequency of intakes of the five protein-rich foods for understanding which sections strongly associate with frailty. The β score of malnutrition was excluded from sections of the KCL because the score was too low to perform the statistical analysis. The frequency of protein-rich food intake was stratified into quartiles, and the prevalence of frailty in relation to the frequency in each quartile was compared with the frequency in the first quartile as a reference; the adjusted means of each quartile were indicated by analysis of covariance. For a robustness check, we used the original KCL score cutoff, 7, for frailty in the same way among the 5750 participants (2822 men and 2928 women) who had fully responded to the KCL. The analysis was performed for men and women to explore any sex differences in relation to frailty. Statistical significance was set as a two-sided *p*-value of 0.05. The statistical analyses were performed using STATA, version 14.0 (StataCorp LP, College Station, TX, USA).

## 3. Results

The pairwise correlation coefficient for assessing the test-retest reproducibility of frequency of protein-rich food intake was 0.54–0.63 in men and 0.42–0.66 in women (Table 1). The foods with the highest frequency of intake among both sexes were seafood and soy products (Table 2). Further, the ROC curve analysis with a total β score of 3.80 as the cutoff point for frailty resulted in an area under the curve (AUC) of 0.86 (95% CI 0.85, 0.86), sensitivity of 89.4%, and specificity of 81.6%. The ROC curve analysis using a score of 7 as the cutoff point in the original KCL resulted in an AUC of 0.82 (95% CI 0.81, 0.83), sensitivity of 96.6%, and specificity of 67.9%.

Table 3 shows the sociodemographic characteristics of the participants divided by frailty among men and women. The average age (years) in the cases of frailty was significantly higher than in the non-frail individuals (frailty: 76.9, SD 7.0 in men, 79.1 SD 6.8 in women; non-frailty: 72.8, SD 5.7 in men, 72.7, SD 5.6 in women). Except for population density, the proportion of each variable varied significantly by frailty (*p*-value < 0.05).

Table 4 presents the adjusted prevalence ratio (PR) for frailty in each quartile of protein-rich food intake frequency compared to the first quartile as a reference, analyzed using the multiple logistic regression model. In Model 2, the highest quartile of intake frequency of seafood showed a significantly lower prevalence of frailty than the lowest quartile among men (PR, 0.64; 95% CI, 0.42, 0.99). Although significant associations between the quartiles were not observed, a positive linear trend was significantly shown in the frequency of the consumption of egg in men (*p* for trend = 0.015). Among women, a significantly low PR for frailty was presented in the second quartile of seafood intake frequency (PR, 0.61; 95% CI 0.43, 0.85) and in the third quartile (PR, 0.64; 95% CI, 0.46, 0.91, *p* for trend = 0.088). The third quartile of dairy products was shown to be inversely associated with the prevalence of frailty when compared to the first quartile (PR, 0.69; 95% CI 0.51, 0.92).

Based on the significant results in Table 3, the multiple regression model was used to indicate which six subdomains of the KCL (i.e., IADL, physical strength, oral function, socialization, memory, and mood) had a significant association with frequency of seafood and dairy products intake. The total β score of the KCL is indicated in Table 5 for men and in Table 6 for women. Specifically, the association between seafood consumption frequency and the IADL β score had a significantly negative linear trend with a lower frailty level across quartiles: from the second to fourth quartile in men (*p* for trend = 0.001) (Table 4). The frequency of seafood intake in the second quartile presented inverse associations with the memory β score ((standardized) beta coefficient, −0.030; standard error (SE), 0.011; *p*-value = 0.008). When compared to the first quartile of seafood intake frequency, the mood β score showed a negative linear trend from the second to the fourth quartiles (*p* for trend = 0.016). The frequency of dairy products intake in the second quartile showed a significantly inverse association with the IADL β score (beta coefficient = −0.085, SE 0.040, *p*-value = 0.035).

Among women, the seafood consumption frequency across quartiles, when compared to the first quartile, had significantly inverse associations with the IADL β score (*p* for trend <0.001) (Table 5). The frequency of seafood intake in the fourth quartile had a significantly inverse association with the memory and mood β scores (*p* for trend = 0.001 in memory; *p* for trend = 0.056 in mood). Furthermore, the frequency of dairy products intake from the third to the fourth quartiles had significantly inverse associations with the IADL β score, physical strength, oral function, and memory (*p* for trend = 0.008 in IADL; *p* for trend = 0.001 in physical strength; *p* for trend = 0.003 in oral function; *p* for trend = 0.026 in memory). The present results in Model 1 were similar to the results in Model 1 when analyzed using the same number of participants as Model 2 (2807 men and 2978 women).

The relationship between frequency of protein-rich food intake and the original KCL score of 25 items was same as those using a weighted β score. The results of the analysis using the original KCL score showed similar associations but more statistically significant results (Table 7).

## 4. Discussion

Our main findings suggest that higher consumption frequencies of seafood in both sexes (over 2.1 times/day in the fourth quartile for men; 1.1–2.2 times/day in the third quartile for women) and dairy products in women (1.0–1.9 times/day in the third quartile) resulted in a 0.61–0.69-fold lower prevalence ratio for frailty compared to the lowest intake frequency group in older Japanese adults. Precisely, the associations between the frequencies of food consumption and the frailty subdomains of KCL were as follows. First, a higher seafood intake frequency was found to be a significant element in predicting the non-frailty status in terms of higher IADL, mood, and memory scores in both sexes. Second, a higher frequency of dairy products intake was associated with higher levels of physical strength, IADL, memory, and oral function scores in women.

The consistency of the current result can be confirmed by the reports of several previous studies. A meta-analysis with 21 cohort studies confirmed the effect of fishery products on reducing the risk of cognitive impairment [11]. Another study demonstrated the association between a dietary habit of fish intake and a reduction in the incidence of late-life depression [9]. This was further confirmed by articles that showed the association of physical function and muscle strength with animal protein intake from fish but not meat [3], the association between higher protein intake and a higher level of physical function [7], and the positive relationship between fatty fish intake and grip strength [30]. Other reports revealed the association of frailty in relation to the Mediterranean diet, in which fish is a major protein source in other components (i.e., olive oil, vegetables, legumes, fruits, and cereal) [31,32]. To our knowledge, this is the first time a dose-response relationship between the frequent consumption of seafood and a higher level of IADL has been described. The mechanism of these relationships can be assumed based on several nutrients from fish interacting with each other leading to physiological strengths, specifically, amino acids for muscle synthesis [3] and polyunsaturated fatty acids for cognition and mood [9,33,34]. Based on the non-significance of the association of physical function with frequent seafood intake in the current result, higher consumption frequencies of seafood may be related to complex behavior for the independence of IADL (e.g., use of public transportation, shopping, and money management) rather than the simple association of neurological function and muscle strength. The significant association between frailty and seafood intake but not meat intake, among major sources of animal protein, is considered as follows. The low frequency of meat intake may affect the null association with the prevalence of frailty. The frequency of seafood intake was higher than that of meat in the present study. A Japanese study among older women also indicated a similar trend of seafood and meat intake, and total animal protein intake was inversely associated with the prevalence of frailty [35]. Therefore, dietary habits of older Japanese people might affect the present association.

Dairy products have been known to be substantial sources of not only protein but also vitamins and minerals for older adults [36,37]. Due to the presence of these nutrients, it has been reported that dairy products have several effects on the mitigation of frailty among older people. Community-dwelling residents aged 60 years or older who consumed seven or more servings per week of low-fat milk and yogurt had a lower incidence of frailty as compared to those who consumed less than one serving per week of these dairy products in Spain [8]. A previous report provides evidence that dietary patterns with a high load of dairy products reduced the risk of the onset of dementia [38]. Recent studies also confirmed the association between higher intakes of dairy products and a lower prevalence of periodontitis among adults and older adults [6,10], whereas a higher intake of dairy products indicated a higher risk of functional disability, but this was not of significance among older Japanese adults [39]. The dairy products in the previous study comprised high-fat foods (i.e., cheese and butter), and this might lead to an inconsistent association with the present study in terms of the results [39].

We should note that in the present study the linear trend between frequencies of seafood in men and women and dairy products in women in relation to the prevalence of frailty did not show significant association except for components of the KCL. The holistic assessment of frailty may be statistically unstable to show the dose-response association of seafood and dairy product frequencies.

This study did not clarify the reason that a higher frequency of egg intake was associated with a significant linear trend of high prevalence of frailty in men. Since of the frequency of egg intake was the lowest among the other protein-rich foods, the impact of the high frequency of egg intake on frailty is unclear. Some latent factors may exist, such as eating other food with egg or the method of cooking.

The present results indicated sex-related differences. Although a part of the groups of quartiles showed a significant association between seafood intake and frailty in both sexes, the significance was observed for a lower frequency of seafood intake in women than men. Further, the significant association between dairy product intake in the third quartiles and frailty was observed in women, but not in men. According to the previous article, women have high rates of depression and anxiety [40]. In addition, older women are more likely to have an increased overall adiposity, which may lead to skeletal muscle damage and low grip strength [40,41]. The sex-related difference in the present results was suspected to be related to the protective effects of seafood and dairy products against frailty symptoms, especially in women.

The objective in using a weighted β score was to assess the KCL score including missing data. Of note, the weighted β score and the cutoff point did not indicate the generalizable value and would change among study populations. However, the process in calculating the weighted β score and the decision on the cutoff point may be available with data of long-term care or some related variables.

This study has several strengths: first, the weighted score of the KCL was calculated using the precise information on the LTC certification in community-dwelling residents. Second, the selection bias was attenuated since the weighted score of the KCL was calculated considering the missing data. As described above, the weighted score of the KCL improved the specificity to predict LTC needs when compared to the original KCL score.

Despite several strengths, the present study has some limitations. First, there was approximately a 6-month time lag between the responses to the KCL at baseline and the FFQ at the additional survey. However, the respondents confirmed their frequency of protein-rich food intake on average during the last year. Consequently, the data were available to enable the merging of the FFQ at the additional survey with the KCL at baseline. Second, the present data were obtained from a self-administered mail survey questionnaire. However, the KCL had certified the validation [18,19], and the study-specific score of the KCL was confirmed by the sensitivity and specificity in the present study. Third, the present participants tended to be both healthy and financially secure compared with people who declined to participate in the additional survey. The mean of the total KCL score (mean = 3.5, SD 2.4) in participants who only responded at baseline was higher than that in participants who responded to the additional survey (mean = 2.0, SD 1.7). This might have caused selection bias and underestimated the association between protein-rich food intake and frailty. Fourth, we did not have information about whether participants received a meal service or others privately other than the national insurance services. The private use status of a meal service may lead to residual confounding. Finally, the present findings, derived from a cross-sectional study, do not necessarily indicate causality. To minimize the possibility of reverse causality, we recruited older adults to our study who were not receiving LTC at baseline.

With respect to the association between the frequency of protein-rich food intake and frailty, further studies are required to clarify the nutritional content of these foods. Milk proteins (i.e., casein and whey) in dairy products are associated with muscle protein synthesis [42], and the quality of fat in seafood (i.e., n-3 long-chain polyunsaturated fatty acid) is associated with recognition and mood [9,33,34].

## 5. Conclusions

This study suggests the possibility that higher frequencies of seafood intake in men and women, and higher intakes of dairy products in women, have a beneficial effect on the prevention of frailty as compared to the lowest frequency group, but not a dose-response association, among older Japanese adults. The current results provide insights into the expectation that an adequate intake of protein-rich food, such as seafood and dairy products, may support older adults in maintaining their independence.

## Figures and Tables

**Table 1:** Test-retest reproducibility of the frequency of protein-rich food intakes assessed by the present food frequency questionnaire in 227 older men and 221 older women.

Food Frequency (Times/Day)	Men					Women				
		Survey 1	Survey 2	*p*-Value	*r*		Survey 1	Survey 2	*p*-Value	*r*
	*n*	Mean ± SD				*n*	Mean ± SD			
Animal food										
Seafood	190	1.70 ± 1.06	1.65 ± 1.07	0.529	0.54^*^	180	1.89 ± 1.26	1.67 ± 0.88	0.018	0.42^*^
Meat	201	0.90 ± 0.65	0.97 ± 0.76	0.106	0.63^*^	194	0.95 ± 0.56	0.98 ± 0.54	0.519	0.54^*^
Dairy products	187	0.86 ± 0.77	1.00 ± 0.86	0.012	0.56^*^	187	1.20 ± 0.92	1.33 ± 0.97	0.024	0.66^*^
Egg	219	0.58 ± 0.44	0.56 ± 0.39	0.301	0.54^*^	217	0.59 ± 0.36	0.59 ± 0.37	0.879	0.53^*^
Soy products	197	1.76 ± 1.21	1.62 ± 1.15	0.074	0.56^*^	189	1.98 ± 1.12	1.93 ± 1.30	0.533	0.57^*^

First survey was conducted in February 2012 and second survey was conducted in September 2012. SD = standard deviation; *r* = correlation coefficients assessed by pairwise correlation coefficient; the differences between Frequency 1 and Frequency 2 assessed by the paired t-test; * *p*-value < 0.001.

**Table 2:** Frequency of intake of protein-rich food among older men and women.

Frequency of Intake	Men		Women	
(Times/Day)	*n*	Median (IQR)	*n*	Median (IQR)
Animal food ^a^				
Seafood	3303	1.4 (0.9, 2.1)	3592	1.6 (1.1, 2.3)
Meat	3461	0.7 (0.5, 1.2)	3805	0.8 (0.5, 1.2)
Dairy products	3292	0.9 (0.1, 1.2)	3677	1.0 (0.4, 2.0)
Egg	3778	0.5 (0.2. 0.8)	4242	0.5 (0.2, 1.0)
Soy products ^b^	3360	1.4 (0.8, 2.2)	3756	1.6 (1.1, 2.5)

IQR = interquartile range (25th percentile, 75th percentile); ^a^ Seafood comprised contained fish, bone-edible small fish, canned tuna, cuttlefish, squid, octopus, shrimp, crab, shellfish, and fish paste products; meat comprised chicken, beef, pork, liver, ham, sausage, bacon, and salami-sausage; dairy products comprised milk and yogurt; and ^b^ soy products comprised miso soup, tofu (soybean curd), fried tofu paste, and fried tofu.

**Table 3:** Sociodemographic characteristics of older men and women.

	Men			Women		
	Non-Frailty (*n* = 3381)	Frailty (*n* = 462)		Non-Frailty (*n* = 3567)	Frailty (*n* = 764)	
	Mean ± SD or *n* (%)		*p*-Value	Mean ± SD or *n* (%)		*p*-Value
Total score of frailty	1.46 ± 1.05	5.03 ± 1.06	-	1.51 ± 1.07	5.09 ± 1.02	-
Age (years)	72.8 ± 5.7	76.9 ±7.0	<0.001 **	72.7 ± 5.6	79.1 ± 6.8	<0.001 **
65–69	1217 (36.0)	85 (18.4)	<0.001 **	1284 (36.0)	78 (10.2)	<0.001 **
70–74	1000 (29.6)	107 (23.2)		1064 (29.8)	122 (16.0)	
75–79	682 (20.2)	97 (21.0)		745 (20.9)	197 (25.8)	
80+	482 (14.3)	173 (37.5)		474 (13.3)	367 (48.0)	
Family structure						
Alone	218 (6.5)	24 (5.2)	0.001 *	575 (16.1)	120 (15.7)	<0.001 **
With someone/others	2936 (86.8)	386 (83.6)		2735 (76.7)	536 (70.2)	
Unknown	227 (6.7)	52 (11.3)		257 (7.2)	108 (14.1)	
Educational attainment (years)						
≤9	790 (23.4)	147 (31.8)	<0.001 **	873 (24.5)	256 (33.5)	<0.001 **
10–12	1314 (38.9)	141 (30.5)		1708 (47.9)	311 (40.7)	
13+	933 (27.6)	81 (17.5)		608 (17.1)	57 (7.5)	
Unknown	344 (10.2)	93 (20.1)		378 (10.6)	140 (18.3)	
Economic situation						
Good	1169 (34.6)	97 (21.0)	<0.001 **	1199 (33.6)	223 (29.2)	0.001 *
Poor	2100 (62.1)	348 (75.3)		2170 (60.8)	477 (62.4)	
Unknown	112 (3.3)	17 (3.7)		198 (5.6)	64 (8.4)	
Dietary supplement use						
Yes	1896 (56.1)	306 (66.2)	<0.001 **	1687 (47.3)	417 (54.6)	<0.001 **
No	1443 (42.7)	141 (30.5)		1811 (50.8)	316 (41.4)	
Unknown	42 (1.2)	15 (3.3)		69 (1.9)	31 (4.1)	
Diet treatment						
Yes	2524 (74.7)	315 (68.2)	<0.001 **	2919 (81.8)	577 (75.5)	<0.001 **
No	815 (24.1)	132 (28.6)		589 (16.5)	166 (21.7)	
Unknown	42 (1.2)	15 (3.3)		59 (1.7)	21 (2.8)	
Current smoking						
Yes	612 (18.1)	85 (18.4)	<0.001 **	124 (3.5)	27 (3.5)	<0.001 **
Past	1954 (57.8)	246 (53.3)		219 (6.1)	51 (6.7)	
Never	714 (21.1)	92 (19.9)		3067 (86.0)	612 (80.1)	
Unknown	101 (3.0)	39 (8.4)		157 (4.4)	74 (9.7)	
BMI (kg/m^2^)	23.0 ± 3.1	22.6 ± 3.9	0.022 *	22.3 ± 3.5	22.8 ± 4.9	0.025 *
<18.5	142 (4.2)	40 (8.7)	<0.001 **	339 (9.5)	92 (12.0)	<0.001 **
18.5–24.9	2420 (71.6)	308 (66.7)		2489 (69.8)	400 (52.4)	
25.0+	692 (20.5)	73 (15.8)		576 (16.2)	151 (19.8)	
Unknown	127 (3.8)	41 (8.9)		163 (4.6)	121 (15.8)	
Total energy intake (kcal/day)	1924 ± 504	1854 ± 516	0.005 *	1573 ± 331	1530 ± 360	0.003 *
Daily physical exercise ^a^						
Yes	1094 (32.4)	228 (49.4)	<0.001 **	1240 (34.8)	419 (54.8)	<0.001 **
No	2139 (63.3)	182 (39.4)		2179 (61.0)	265 (34.7)	
Unknown	148 (4.4)	52 (11.3)		148 (4.2)	80 (10.5)	
Population density (people/km^2^)						
Province	1539 (45.4)	215 (46.5)	0.680	1661 (46.6)	339 (44.4)	0.270
City	1842 (54.5)	247 (53.5)		1906 (53.4)	425 (55.6)	

SD = standard deviation; BMI = body mass index; population density was divided by 1000 people/km^2^; The difference between non-frailty and frailty was analyzed using the chi-square test for categorical variables and the unpaired t-test for continuous variables; ^a^ Daily physical exercise included walking and other physical exercises; * *p*-value <0.05; ** *p*-value <0.001.

**Table 4:** Prevalence ratio (PR) and 95% confidence interval (CI) for frailty assessed by the weighted β score of the Kihon-Checklist in the quartile of protein-rich food intake frequency compared with the first quartile analyzed using multiple logistic regression among older men and women.

	Q1 (Lowest)	Q2		Q3		Q4 (Highest)		*p* for Trend
	PR (95% CI)	PR (95% CI)	*p*-Value	PR (95% CI)	*p*-Value	PR (95% CI)	*p*-Value	
Men								
Animal food (times/day)								
Seafood								
Case/total *n* (%)	99/741 (13.4)	87/917 (9.5)		86/843 (10.2)		82/802 (10.2)		
Model 1	Reference	0.72 (0.52, 1.00)	0.049 *	0.77 (0.55, 1.08)	0.135	0.70 (0.50, 0.99)	0.046 *	
Model 2	Reference	0.70 (0.48, 1.03)	0.070	0.76 (0.51, 1.14)	0.181	0.64 (0.42, 0.99)	0.046 *	0.086
Meat								
Case/total *n* (%)	99/841 (11.8)	96/971 (9.9)		87/756 (11.5)		97/893 (10.9)		
Model 1	Reference	0.95 (0.69, 1.31)	0.764	1.11 (0.80, 1.55)	0.524	1.02 (0.74, 1.41)	0.892	
Model 2	Reference	1.13 (0.76, 1.68)	0.535	1.42 (0.95, 2.13)	0.091	1.19 (0.79, 1.81)	0.404	0.274
Dairy products								
Case/total *n* (%)	100/717 (14.0)	99/917 (10.8)		76/780 (9.7)		102/881 (11.6)		
Model 1	Reference	1.02 (0.74, 1.40)	0.923	0.99 (0.70, 1.41)	0.973	1.34 (0.96, 1.88)	0.086	
Model 2	Reference	0.86 (0.59, 1.25)	0.422	0.84 (0.56, 1.26)	0.403	1.40 (0.96, 2.06)	0.084	0.098
Egg								
Case/total *n* (%)	54/418 (12.9)	99/989 (10.0)		113/1068 (10.6)		180/1303 (13.8)		
Model 1	Reference	0.80 (0.55, 1.17)	0.249	0.81 (0.56, 1.18)	0.280	1.10 (0.77, 1.57)	0.607	
Model 2	Reference	0.82 (0.50, 1.34)	0.427	0.78 (0.47, 1.29)	0.335	1.41 (0.87, 2.27)	0.160	0.015 *
Soy products (times/day)								
Case/total *n* (%)	88/682 (12.9)	108/1061 (10.2)		76/682 (11.1)		104/935 (11.1)		
Model 1	Reference	0.77 (0.55, 1.06)	0.109	0.87 (0.61, 1.24)	0.438	0.76 (0.54, 1.08)	0.123	
Model 2	Reference	0.83 (0.57, 1.21)	0.343	0.84 (0.55, 1.29)	0.422	0.81 (0.53, 1.22)	0.308	0.374
Women								
Animal food (times/day)								
Seafood								
Case/total *n* (%)	188/877 (21.4)	122/881 (13.9)		137/1006 (13.6)		126/828 (15.2)		
Model 1	Reference	0.64 (0.48, 0.86)	0.003 *	0.62 (0.46, 0.82)	0.001 *	0.65 (0.48, 0.89)	0.007 *	
Model 2	Reference	0.61 (0.43, 0.85)	0.004 *	0.64 (0.46, 0.91)	0.013 *	0.70 (0.48, 1.02)	0.066	0.088
Meat								
Case/total *n* (%)	161/761 (21.2)	153/977 (15.7)		134/922 (14.5)		177/1145 (15.5)		
Model 1	Reference	0.86 (0.64, 1.14)	0.287	0.83 (0.62, 1.11)	0.215	0.86 (0.64, 1.14)	0.281	
Model 2	Reference	0.81 (0.57, 1.15)	0.236	0.89 (0.62, 1.26)	0.504	0.90 (0.63, 1.28)	0.560	0.759
Dairy products								
Case/total *n* (%)	208/902 (23.1)	76/465 (16.3)		194/1382 (14.0)		130/928 (14.0)		
Model 1	Reference	0.86 (0.61, 1.20)	0.369	0.71 (0.55, 0.92)	0.008 *	0.78 (0.59, 1.04)	0.096	
Model 2	Reference	0.86 (0.59, 1.27)	0.457	0.69 (0.51, 0.92)	0.013 *	0.90 (0.64, 1.27)	0.549	0.168
Egg								
Case/total *n* (%)	85/380 (22.4)	193/1040 (18.6)		268/1736 (15.4)		192/1086 (17.7)		
Model 1	Reference	0.92 (0.66, 1.29)	0.620	0.78 (0.57, 1.08)	0.133	0.84 (0.60, 1.18)	0.314	
Model 2	Reference	1.00 (0.65, 1.55)	0.987	0.90 (0.60, 1.37)	0.636	0.81 (0.51, 1.26)	0.348	0.217
Soy products (times/day)								
Case/total *n* (%)	181/897 (20.2)	130/862 (15.1)		160/1036 (15.4)		154/961 (16.0)		
Model 1	Reference	0.74 (0.56, 0.99)	0.043 *	0.80 (0.61, 1.05)	0.103	0.75 (0.57, 1.00)	0.051	
Model 2	Reference	0.84 (0.60, 1.18)	0.318	0.94 (0.67, 1.31)	0.713	0.95 (0.67, 1.36)	0.800	0.924

Q = quartile of frequencies of protein-rich food; Q1: <25th percentile; Q2: ≥25th and <50th; Q3: ≥50th and <75th; Q4: ≥75th; Model 1, a fully adjusted multiple logistic regression model, used age (years), family structure (alone, with someone/others, and unknown), educational attainment (≤9, 10–12, ≥13 years, and unknown), self-rated economic conditions (good, poor, and unknown), diet supplement use (yes, no, and unknown), diet treatment (yes, no, and unknown), smoking habits (yes, past, never, and unknown), body mass index (<18.5, 18.5–24.9, and ≥25 kg/m^2^, and unknown), total energy intake (kcal/day), and population density (≥1000 or <1000 people/km^2^). Model 2, Model 1+ all groups of food frequencies (e.g., seafood, meat, dairy product, egg, and soy product) among 2807 men and 2978 women; *p* for trend, the linear trend test across the quartile groups of food intake frequency was performed; * *p*-value < 0.05.

**Table 5:** The multiple regression model for the associations between the frequency of seafood and dairy products and the six subdomains of the Kihon-Checklist for assessing frailty among older men.

	**Frequency of Seafood Intake**							***p*** **for Trend**
	**Q1 (Lowest)**	**Q2**	***p*-Value**	**Q3**	***p*-value**	**Q4 (Highest)**	***p*-Value**	
*n*	741	917		843		802		
IADL								
Means (SE)	0.76 (0.031)	0.62 (0.026)		0.63 (0.028)		0.60 (0.031)		
*β* (SE)	Reference	−0.137 (0.040)	0.001 *	−0.130 (0.043)	0.002 *	−0.169 (0.046)	<0.001 **	0.001 *
Physical function								
Means (SE)	0.70 (0.033)	0.65 (0.028)		0.68 (0.029)		0.70 (0.033)		
*β* (SE)	Reference	−0.052 (0.043)	0.228	−0.030 (0.045)	0.514	−0.015 (0.049)	0.765	0.945
Oral function								
Means (SD)	0.08 (0.005)	0.07 (0.004)		0.07 (0.004)		0.07 (0.005)		
*β* (SE)	Reference	−0.010 (0.006)	0.094	−0.009 (0.006)	0.168	−0.013 (0.007)	0.056	0.093
Socialization								
Means (SE)	0.04 (0.004)	0.04 (0.003)		0.04 (0.003)		0.04 (0.004)		
*β* (SE)	Reference	−0.0003 (0.005)	0.949	−0.004 (0.005)	0.501	−0.006 (0.006)	0.262	0.204
Memory								
Means (SE)	0.16 (0.009)	0.13 (0.007)		0.14 (0.008)		0.14 (0.008)		
*β* (SE)	Reference	−0.030 (0.011)	0.008 *	−0.020 (0.012)	0.087	−0.014 (0.013)	0.267	0.500
Mood								
Means (SE)	0.19 (0.008)	0.16 (0.007)		0.16 (0.007)		0.16 (0.008)		
*β* (SE)	Reference	−0.031 (0.011)	0.005 *	−0.031 (0.012)	0.008 *	−0.033 (0.012)	0.008 *	0.016 *
	**Frequency of Dairy Products Intake**							***p*** **for Trend**
	**Q1 (Lowest)**	**Q2**	***p*-Value**	**Q3**	***p*-Value**	**Q4 (Highest)**	***p*-Value**	
*n*	717	917		780		878		
IADL								
Means (SE)	0.71 (0.030)	0.61 (0.026)		0.64 (0.028)		0.65 (0.028)		
*β* (SE)	Reference	−0.085 (0.040)	0.035 *	−0.060 (0.042)	0.149	−0.051 (0.042)	0.228	0.433
Physical function								
Means (SE)	0.70 (0.032)	0.66 (0.028)		0.64 (0.030)		0.73 (0.029)		
*β* (SE)	Reference	−0.028 (0.043)	0.503	−0.051 (0.044)	0.253	0.044 (0.045)	0.328	0.355
Oral function								
Means (SD)	0.08 (0.005)	0.07 (0.004)		0.07 (0.004)		0.07 (0.004)		
*β* (SE)	Reference	−0.009 (0.006)	0.120	−0.010 (0.006)	0.098	−0.008 (0.006)	0.183	0.233
Socialization								
Means (SE)	0.04 (0.004)	0.04 (0.003)		0.03 (0.003)		0.05 (0.003)		
*β* (SE)	Reference	0.003 (0.005)	0.554	−0.005 (0.005)	0.349	0.010 (0.005)	0.062	0.170
Memory								
Means (SE)	0.13 (0.008)	0.14 (0.007)		0.15 (0.008)		0.14 (0.008)		
*β* (SE)	Reference	0.003 (0.011)	0.788	0.012 (0.011)	0.309	0.006 (0.012)	0.608	0.483
Mood								
Means (SE)	0.16 (0.008)	0.17 (0.007)		0.16 (0.008)		0.17 (0.007)		
*β* (SE)	Reference	0.009 (0.011)	0.382	0.002 (0.011)	0.916	0.011 (0.011)	0.334	0.513

IADL = instrumental activities of daily living; Means (SE) = fully adjusted means (standard error) of food frequency (times/day) by analysis of covariance; *β* (SE) = standardized (beta) coefficient (standard error); Q = quartile of frequencies of protein-rich food; Q1: <25th percentile; Q2: ≥25th and <50th; Q3: ≥50th and <75th; Q4: ≥75th; the regression model was used adjusting for age (years), family members (alone, with someone/others, and unknown), educational attainment (<9, 10–12, ≥13 years, and unknown), nutritional supplement use (yes, no, and unknown), dietary treatment (yes, no, and unknown), smoking habits (yes, no, and unknown), body mass index (<18.5, 18.5–24.9, ≥25 kg/m^2^, and unknown), total energy intake (kcal/day), and population density (≥1000 or <1000 people/km^2^) in addition to all the groups of food intake frequencies (e.g., seafood, meat, dairy product, egg, and soy product) among 2807 men; *p* for trend, the linear trend test across the quartile groups of the food intake frequency was performed; * *p*-value < 0.05; ** *p*-value < 0.001.

**Table 6:** The multiple regression model for the associations between the frequency of seafood and dairy products and the six subdomains of the Kihon-Checklist for assessing frailty among older women.

	**Frequency of Seafood Intake**							***p*** **for Trend**
	**Q1 (Lowest)**	**Q2**	***p*-Value**	**Q3**	***p*-Value**	**Q4 (Highest)**	***p*-Value**	
*n*	877	881		1006		828		
IADL								
Means (SE)	0.62 (0.027)	0.48 (0.025)		0.48 (0.023)		0.43 (0.028)		
*β* (SE)	Reference	−0.132 (0.035)	<0.001 **	−0.135 (0.036)	<0.001 **	−0.183 (0.041)	<0.001 **	<0.001 **
Physical function								
Means (SE)	1.07 (0.035)	1.00 (0.032)		1.07 (0.030)		1.10 (0.036)		
*β* (SE)	Reference	−0.065 (0.046)	0.158	0.002 (0.047)	0.961	0.031 (0.053)	0.557	0.340
Oral function								
Means (SD)	0.08 (0.004)	0.08 (0.004)		0.07 (0.004)		0.07 (0.005)		
*β* (SE)	Reference	−0.002 (0.006)	0.791	−0.005 (0.006)	0.362	−0.011 (0.007)	0.093	0.080
Socialization								
Means (SE)	0.05 (0.004)	0.04 (0.003)		0.05 (0.003)		0.05 (0.004)		
*β* (SE)	Reference	−0.001 (0.005)	0.879	0.005 (0.005)	0.286	0.0002 (0.006)	0.967	0.642
Memory								
Means (SE)	0.14 (0.008)	0.13 (0.007)		0.12 (0.007)		0.09 (0.008)		
*β* (SE)	Reference	−0.012 (0.010)	0.255	−0.013 (0.010)	0.206	−0.042 (0.012)	<0.001 **	0.001 *
Mood								
Means (SE)	0.20 (0.009)	0.18 (0.008)		0.19 (0.008)		0.16 (0.009)		
*β* (SE)	Reference	−0.018 (0.012)	0.133	−0.007 (0.012)	0.543	−0.032 (0.013)	0.017 *	0.056
	**Frequency of Dairy Products Intake**							***p*** **for Trend**
	**Q1 (Lowest)**	**Q2**	***p*-Value**	**Q3**	***p*-Value**	**Q4 (Highest)**	***p*-Value**	
*n*	902	465		1382		928		
IADL								
Means (SE)	0.56 (0.025)	0.47 (0.034)		0.49 (0.020)		0.47 (0.026)		
*β* (SE)	Reference	−0.061 (0.042)	0.146	−0.076 (0.032)	0.017 *	−0.093 (0.037)	0.011 *	0.008 *
Physical function								
Means (SE)	1.16 (0.033)	1.05 (0.043)		1.01 (0.025)		1.04 (0.033)		
*β* (SE)	Reference	−0.079 (0.054)	0.143	−0.152 (0.041)	<0.001 **	−0.136 (0.047)	0.004 *	0.001 *
Oral function								
Means (SD)	0.08 (0.004)	0.08 (0.005)		0.07 (0.003)		0.07 (0.004)		
*β* (SE)	Reference	−0.003 (0.007)	0.713	−0.012 (0.005)	0.022 *	−0.015 (0.006)	0.010 *	0.003 *
Socialization								
Means (SE)	0.04 (0.034)	0.05 (0.004)		0.05 (0.003)		0.05 (0.003)		
*β* (SE)	Reference	0.010 (0.006)	0.088	0.006 (0.004)	0.199	0.003 (0.005)	0.570	0.613
Memory								
Means (SE)	0.14 (0.007)	0.12 (0.010)		0.11 (0.006)		0.12 (0.007)		
*β* (SE)	Reference	−0.016 (0.012)	0.186	−0.023 (0.009)	0.012 *	−0.021 (0.011)	0.049 *	0.026 *
Mood								
Means (SE)	0.18 (0.008)	0.19 (0.011)		0.18 (0.006)		0.19 (0.008)		
*β* (SE)	Reference	0.010 (0.014)	0.446	-0.005 (0.011)	0.654	0.008 (0.012)	0.513	0.838

Means (SE) = fully adjusted means (standard error) of food frequency (times/day) by analysis of covariance; *β* (SE) = standardized (beta) coefficient (standard error); Q = quartile of frequencies of protein-rich food; Q1: ≤25th percentile; Q2: ≥25th and <50th; Q3: ≥50th and <75th; Q4: ≥75th; The regression model was used adjusting for age (years), family members (alone, with someone/others, and unknown), educational attainment (<9, 10–12, ≥13 years, and unknown), nutritional supplement use (yes, no, and unknown), dietary treatment (yes, no, and unknown), smoking habits (yes, no, and unknown), body mass index (<18.5, 18.5–24.9, ≥25 kg/m^2^, and unknown), total energy intake (kcal/day), and population density (≥1000 or <1000 people/km^2^) in addition to all the groups of food intake frequencies (e.g., seafood, meat, dairy product, egg, and soy product) among 2978 women; *p* for trend, the linear trend test across the quartile groups of the food intake frequency was performed; * *p*-value < 0.05; ** *p*-value < 0.001.

**Table 7:** Prevalence ratio (PR) and 95% confidence interval (CI) for frailty assessed by the original 25-item Kihon-Checklist in the quartile of protein-rich food intake frequency compared with the first quartile analyzed using multiple logistic regression among 2822 older men and 2928 older women.

	Q1 (Lowest)	Q2		Q3		Q4 (Highest)		*p* for Trend
	PR (95% CI)	PR (95% CI)	*p*-Value	PR (95% CI)	*p*-Value	PR (95% CI)	*p*-Value	
Men								
Animal food (times/day)								
Seafood								
Case/total *n* (%)	215/626 (34.4)	154/654 (23.6)		206/661 (31.2)		153/587 (26.1)		
Model 1	Reference	0.55 (0.42, 0.72)	<0.001 **	0.84 (0.65, 1.09)	0.199	0.58 (0.44, 0.77)	<0.001 **	
Model 2	Reference	0.54 (0.40, 0.73)	<0.001 **	0.87 (0.64, 1.16)	0.337	0.61 (0.44, 0.85)	0.004 *	0.065
Meat								
Case/total *n* (%)	186/610 (30.5)	221/746 (29.6)		174/596 (29.2)		174/666 (26.1)		
Model 1	Reference	1.09 (0.84, 1.40)	0.517	1.01 (0.77, 1.32)	0.950	0.89 (0.68, 1.16)	0.382	
Model 2	Reference	1.13 (0.84, 1.51)	0.417	1.05 (0.77, 1.44)	0.741	0.84 (0.61, 1.16)	0.285	0.219
Dairy products								
Case/total *n* (%)	184/528 (34.9)	224/716 (31.3)		145/588 (24.7)		166/656 (25.3)		
Model 1	Reference	1.04 (0.80, 1.36)	0.762	0.78 (0.58, 1.03)	0.083	0.86 (0.65, 1.14)	0.291	
Model 2	Reference	1.09 (0.82, 1.45)	0.565	0.82 (0.60, 1.11)	0.202	0.92 (0.67, 1.25)	0.579	0.244
Egg								
Case/total *n* (%)	94/299 (31.4)	204/753 (27.1)		230/786 (29.3)		290/959 (30.2)		
Model 1	Reference	0.84 (0.61, 1.15)	0.280	0.84 (0.61, 1.15)	0.270	0.90 (0.66, 1.22)	0.493	
Model 2	Reference	0.97 (0.66, 1.43)	0.887	1.13 (0.77, 1.66)	0.534	1.31 (0.89, 1.92)	0.175	0.045 *
Soy products (times/day)								
Case/total *n*(%)	162/529 (30.6)	229/810 (28.3)		138/525 (26.3)		203/673 (30.2)		
Model 1	Reference	0.88 (0.68, 1.14)	0.341	0.77 (0.58, 1.04)	0.092	0.87 (0.66, 1.15)	0.318	
Model 2	Reference	1.02 (0.76, 1.36)	0.913	0.83 (0.59, 1.16)	0.273	0.99 (0.72, 1.37)	0.952	0.691
Women								
Animal food (times/day)								
Seafood								
Case/total *n* (%)	233/615 (37.9)	200/655 (30.5)		190/719 (26.4)		313/1053 (29.7)		
Model 1	Reference	0.74 (0.57, 0.97)	0.026 *	0.59 (0.45, 0.77)	<0.001 **	0.51 (0.38, 0.68)	<0.001 **	<0.001 **
Model 2	Reference	0.74 (0.55, 1.00)	0.048 *	0.64 (0.47, 0.86)	0.004 *	0.50 (0.35, 0.71)	<0.001 **	<0.001 **
Meat								
Case/total *n* (%)	181/499 (36.3)	229/708 (32.3)		177/672 (26.3)		227/820 (27.7)		
Model 1	Reference	0.93 (0.71, 1.22)	0.591	0.72 (0.54, 0.96)	0.023 *	0.80 (0.61, 1.05)	0.106	
Model 2	Reference	0.93 (0.68, 1.27)	0.645	0.73 (0.53, 1.02)	0.063	0.89 (0.64, 1.23)	0.479	0.324
Dairy products								
Case/total *n* (%)	245/607 (40.4)	100/319 (31.4)		280/1021 (27.5)		154/657 (23.4)		
Model 1	Reference	0.85 (0.62, 1.17)	0.329	0.67 (0.53, 0.86)	0.001 *	0.60 (0.46, 0.80)	<0.001 **	
Model 2	Reference	0.92 (0.65, 1.32)	0.657	0.71 (0.54, 0.93)	0.013	0.65 (0.47, 0.89)	0.008 *	0.002 *
Egg								
Case/total *n* (%)	97/246 (39.4)	223/710 (31.4)		236/900 (26.2)		336/1044 (32.2)		
Model 1	Reference	0.82 (0.58, 1.15)	0.250	0.65 (0.46, 0.91)	0.011*	0.86 (0.62, 1.20)	0.382	
Model 2	Reference	1.12 (0.74, 1.71)	0.584	0.80 (0.53, 1.21)	0.295	1.25 (0.83, 1.88)	0.293	0.340
Soy products (times/day)								
Case/total *n* (%)	235/645 (36.4)	187/613 (30.5)		189/683 (27.7)		202/736 (27.5)		
Model 1	Reference	0.82 (0.63, 1.07)	0.145	0.77 (0.59, 1.00)	0.053	0.70 (0.54, 0.91)	0.009 *	
Model 2	Reference	0.89 (0.66, 1.20)	0.452	0.94 (0.69, 1.27)	0.675	0.79 (0.57, 1.08)	0.142	0.200

Q = quartile of frequencies of protein-rich food; Q1: <25th percentile; Q2: ≥25th and <50th; Q3: ≥50th and <75th; Q4: ≥75th; adjusting variables are same as Table 4. Model 1, a fully adjusted multiple logistic regression model, used age, family structure, educational attainment, self-rated economic conditions, diet supplement use, diet treatment, smoking habits, body mass index, total energy intake, and population density. Model 2, Model 1+ all groups of food frequencies (e.g., seafood, meat, dairy product, egg, and soy product) among 2189 men and 2210 women; *p* for trend, the linear trend test across the quartile groups of food intake frequency was performed; * *p*-value < 0.05; ** *p*-value < 0.001.

## Appendix A

Definition of frailty using a regression model as a conditional mean imputation.

To make the Kihon-Checklist (KCL) score correspond with the predicted missing-KCL score, the weighted KCL score was simultaneously assessed in the present study. The frailty-risk point (+1) was weighted from the beta (β) coefficient using binomial logistic regression with the baseline data (12,054 people without long-term care (LTC), and 1240 people with LTC). We used 25 items of the KCL as explanatory variables with missing variables. The models were as follows:

(1) $$\mathrm{logit}\mathrm{Pr}\;\left(\mathrm{LTC}\;\mathrm{certification}\right)=b_{0}+\beta_{i\;risk}KCL_{i\;risk}+\beta_{i\;miss}KCL_{i\;miss}+\varepsilon$$

Here, i risk (range 1–25) is modeled by the risk variable of the KCL and i miss is modeled by the missing variable of the KCL with the intercept (b0) and the residual (ε). β represents a standardized beta coefficient, and was manipulated the weight to the risk point (+1) of KCL. The score indicates that if the item has a risk of frailty or is missing, the probability of long-term care certification would be raised by β. A score of zero suggests no risk of frailty if the β indicates the inverse association with LTC certification. Thus, the total weighted score of the KCL (total β score) ranged from 0 to 10.69 with 23 items due to the lack of scores for two specific items in the section of ‘oral function’ and ‘mood’ (Table A1). When the total β score was classified with 5 percentiles (i.e., 10th percentile = 0.25, 25th = 0.87, median = 2.02, 75th = 3.80, 90th = 5.95), the 75th percentile (3.80) was shown to be the greatest prediction point against the long-term care certification by the receiver operating characteristic (ROC) curve analysis (area under the curve (AUC) 0.86, 95% CI 0.85, 0.86; sensitivity = 89.4%; specificity = 81.6%). Thus, the cutoff point of 7 in the original KCL was used as a supplementary result of the association between food frequency and frailty in the current study (AUC 0.82, 95% CI 0.81, 0.83; sensitivity = 96.6%; specificity = 67.9%).

## Appendix B

**Table A1:** The weighted *β* score of the Kihon-Checklist (KCL) using the association of long-term care need with 25 items of KCL by the multivariate logistic regression model.

**Kihon-Checklist (KCL)**	**Answer**	***β*** **coefficient (SE)**	***p*****-value**	**β score**	**KCL score**
**Instrumental activities of daily living**				**Range 0–3.65**	**Range 0–5**
1) Do you go out by bus or train by yourself?	Yes	Reference		0	0
	No	0.87 (0.10)	**<0.001**	0.87	1
	Missing	0.16 (0.32)	0.612	0.16	-
2) Do you go shopping to buy daily necessities by yourself?	Yes	Reference		0	0
	No	0.77 (0.11)	**<0.001**	0.77	1
	Missing	1.33 (0.27)	**<0.001**	1.33	-
3) Do you manage your own deposits and saving at the bank?	Yes	Reference		0	0
	No	0.46 (0.10)	**<0.001**	0.46	1
	Missing	-0.19 (0.31)	0.550	0	-
4) Do you sometimes visit your friends?	Yes	Reference		0	0
	No	0.35 (0.10)	**0.001**	0.35	1
	Missing	0.36 (0.30)	0.236	0.36	-
5) Do you turn to your family or friends for advice?	Yes	Reference		0	0
	No	0.63 (0.09)	**<0.001**	0.63	1
	Missing	0.12 (0.26)	0.635	0.12	-
**Physical function/strength**				**Range 0–3.23**	**Range 0–5**
6) Do you normally climb stairs without using any handrails or wall for support?	Yes	Reference		0	0
	No	0.45 (0.12)	**<0.001**	0.45	1
	Missing	0.14 (0.23)	0.532	0.14	-
7) Do you normally stand up from a chair without any aids?	Yes	Reference		0	0
	No	1.20 (0.10)	**<0.001**	1.20	1
	Missing	0.64 (0.33)	0.050	0.64	-
8) Do you normally walk continuously for 15 minutes?	Yes	Reference		0	0
	No	0.50 (0.09)	**<0.001**	0.50	1
	Missing	0.03 (0.30)	0.908	0.03	-
9) Have you experienced a fall in the past year?	Yes	0.25 (0.08)	0.004	0.25	1
	No	Reference		0	0
	Missing	-0.14 (0.23)	0.556	0	-
10) Do you have a fear of falling while walking?	Yes	0.83 (0.12)	**<0.001**	0.83	1
	No	Reference		0	0
	Missing	0.49 (0.22)	0.027	0.49	-
**Malnutrition**				**Range 0–0.34**	**Range 0–2**
11) Have you lost 2kg or more in the past 6 months?	Yes	Reference		0	0
	No	0.04 (0.10)	0.683	0.04	1
	Missing	-0.02 (0.12)	0.883	0	-
12) Body mass index (kg/m^2^) is less than 18.5	Yes	0.12 (0.12)	0.312	0.12	1
	No	Reference		0	0
	Missing	0.30 (0.13)	**0.019**	0.30	-
**Oral function**				**Range 0–0.70**	**Range 0–3**
13) Do you have any difficulties eating tough foods compared to 6 months ago?	Yes	0.21 (0.09)	**0.018**	0.21	1
	No	Reference		0	0
	Missing	0.09 (0.31)	0.775	0.09	-
14) Have you choked on your tea or soup recently?	Yes	-0.15 (0.09)	0.087	0	1
	No	Reference		0	0
	Missing	0.49 (0.32)	0.126	0.49	-
15) Do you often experience having a dry mouth?^a^	Yes	-0.02 (0.09)	0.775	0	1
	No	Reference		0	0
	Missing	-0.11 (0.21)	0.590	0	-
**Kihon-Checklist (KCL)**	**Answer**	***β*** **coefficient (SE)**	***p*****-value**	**β score**	**KCL score**
**Socialization**				**Range 0–0.59**	**Range 0–2**
16) Do you go out at least a week?	Yes	Reference		0	0
	No	-0.41 (0.10)	**<0.001**	0	1
	Missing	0.21 (0.29)	0.464	0.21	-
17) Do you go out less frequently compared to last year?	Yes	0.11 (0.09)	0.246	0.11	1
	No	Reference		0	0
	Missing	0.38 (0.29)	0.185	0.38	-
**Memory**				**Range 0–0.82**	**Range 0–3**
18) Do your family or your friends point out your memory loss? e.g. “You always ask the same question over and over again.”	Yes	0.17 (0.09)	0.066	0.17	1
	No	Reference		0	0
	Missing	0.05 (0.25)	0.854	0.05	-
19) Do you make a call by looking up phone number?	Yes	Reference		0	0
	No	0.29 (0.09)	**0.002**	0.29	1
	Missing	-0.28 (0.32)	0.382	0	-
20) Do you find yourself not knowing today’s date?	Yes	0.36 (0.09)	**<0.001**	0.36	1
	No	Reference		0	0
	Missing	0.26 (0.32)	0.415	0.26	-
**Mood**				**Range 0–1.36**	**Range 0–5**
21) In the last two weeks have you felt lack of fulfillment in your life?	Yes	0.14 (0.10)	0.187	0.14	1
	No	Reference		0	0
	Missing	0.34 (0.22)	0.131	0.34	-
22) In the last two weeks have you felt a lack of joy when doing the things you used to enjoy?^a^	Yes	-0.06 (0.11)	0.575	0	1
	No	Reference		0	0
	Missing	-0.22 (0.23)	0.326	0	-
23) In the last two weeks have you felt difficulty in doing what you could do easily before?	Yes	0.24 (0.11)	**0.031**	0.24	1
	No	Reference		0	0
	Missing	-0.31 (0.27)	0.243	0	-
24) In the last two weeks have you felt helpless?	Yes	0.11 (0.10)	0.271	0.11	1
	No	Reference		0	0
	Missing	-0.14 (0.23)	0.552	0	-
25) In the last two weeks have you felt tired without a reason?	Yes	0.08 (0.11)	0.466	0.08	1
	No	Reference		0	0
	Missing	0.67 (0.27)	**0.012**	0.67	-
				**Total range** **0–10.69**	**Total range** **0–25**

**Foot note of Table A1.** SE = standard error; All variables were adjusted by the multilevel logistic regression model; cutoff point 3.80 in the 75^th^ percentile of the *β* score indicated the area under the curve (AUC), 0.86; 95% confidence interval: 95% CI, 0.85, 0.86; sensitivity, 89.4%; and specificity, 81.6% by receiver operating characteristic (ROC) curve analysis; cutoff point 7 in the KCL score analyzed by ROC curve analysis indicated AUC, 0.82; 95% CI, 0.81, 0.83; sensitivity, 96.6%, and specificity, 67.9%; ^a^ The points of two items were excluded from the *β* score due to the inverse association with the long-term care need.; **bold** *p*-value <0.05
